# Herausforderungen und Perspektiven der Angehörigenbegleitung auf der Intensivstation: Fokus auf vulnerable Angehörige

**DOI:** 10.1007/s00063-025-01262-z

**Published:** 2025-03-18

**Authors:** Magdalena Hoffmann, Matthias Thomas Exl, Maria Brauchle, Karin Amrein, Marie-Madlen Jeitziner

**Affiliations:** 1 Research Unit Safety and Sustainability in Health Care c/o Klinische Abteilung für plastische, ästhetische und rekonstruktive Chirurgie, Universitätsklinik für Chirurgie, Medizinische Universität Graz, Graz, Österreich; 2 Stabsstelle für Qualitäts- und Risikomanagement, LKH-Univ. Klinikum Graz, Graz, Österreich; 3 Universitätsklinik für Neurochirurgie, Universitätsspital Bern (Inselspital), Universität Bern, Bern, Schweiz; 4 Universitätsklinik für Intensivmedizin, Universitätsspital Bern (Inselspital), Universität Bern, Bern, Schweiz; 5 Graduate School for Health Science, University of Bern, Bern, Schweiz; 6 Pflegeschule Vorarlberg, Standort Feldkirch, Vorarlberger Landeskrankenhäuser, Feldkirch, Österreich; 7 Universitätsklinik für Innere Medizin, Abteilung für Endokrinologie und Diabetologie, Medizinische Universität Graz, Graz, Österreich; 8 Institut für Pflegewissenschaft – Nursing Science (INS), Medizinische Fakultät, Departement Public Health (DPH), Universität Basel, Basel, Schweiz; 9 Direktion Pflege INSEL GRUPPE, Universitätsspital Bern (Inselspital), Universität Bern, Bern, Schweiz; 10 Medizinische Universität Graz, Auenbruggerplatz 1/3, 8036 Graz, Österreich

**Keywords:** Kinder, Soziale Unterstützung, Migrant*innen, Coping-Fähigkeiten, Kommunikation, Children, Social support, Immigrants, Coping skills, Communication

## Abstract

**Hintergrund:**

Die Begleitung von Angehörigen auf der Intensivstation (Intensive Care Unit, ICU) stellt eine komplexe Aufgabe dar, insbesondere wenn es sich um vulnerable Menschen handelt wie beispielsweise Kinder, hochaltrige Menschen oder sozioökonomisch benachteiligte Personen. Sie sind besonderen physischen und psychischen Belastungen ausgesetzt und erfordern eine spezifische Expertise des Fachpersonals.

**Fragestellung:**

Welche personenzentrierten Herausforderungen und Unterstützungsangebote gibt es für vulnerable Angehörige auf der Intensivstation?

**Ergebnisse:**

Der vorliegende Artikel beleuchtet die verschiedenen Herausforderungen und Unterstützungsangebote von vulnerablen Angehörigen auf der ICU. Dabei zeigen sich je nach Gruppe und Individuum eine Vielzahl an Herausforderungen wie beispielsweise fehlende Coping-Strategien, erschwerte Zugänglichkeit zu adäquaten Informationen, Kommunikationsprobleme und generell ein erhöhtes Risiko für belastende Langzeitfolgen. Gezielte Schulungen des Fachpersonals, konkrete Praxisanleitungen, kultursensible Kommunikationsstrategien und der Einsatz unterstützender Ressourcen – auch digitaler – können eine personenzentrierte und vulnerabilitätsmindernde Begleitung ermöglichen.

**Schlussfolgerung:**

Eine hohe interprofessionelle und interkulturelle Kompetenz sowie die Entwicklung spezifischer Unterstützungsangebote kann sowohl zur Entlastung der Angehörigen als auch des Fachpersonals führen und zur Verbesserung der Versorgungsqualität beitragen.

## Abstract (en)

**Background:**

Supporting relatives in the intensive care unit (ICU) is a complex task, especially when it comes to vulnerable people such as children, very old persons or socioeconomically disadvantaged persons. They are exposed to particular physical and psychological stress and require targeted awareness from healthcare professionals.

**Objective:**

What person-centered challenges and support services are available for vulnerable relatives in the ICU?

**Results:**

This article highlights the various challenges and support services for vulnerable relatives in ICUs. Depending on the group and individual, there are a variety of challenges such as a lack of helpful coping strategies, difficult access to adequate information, communication problems and a generally increased risk of persistent long-term consequences. Targeted training of specialist staff, specific practical instructions, culturally sensitive communication strategies and the use of supporting resources even digital ones can enable person-centered and vulnerability-reducing support.

**Conclusion:**

A high level of interprofessional and intercultural competence and the development of specific support services can relieve the burden on both relatives and specialist staff and contribute to improving the quality of care.

Eine personenzentrierte multiprofessionelle Begleitung von vulnerablen Angehörigen auf einer Intensivstation (Intensive Care Unit, ICU) stellt eine große Herausforderung für alle am Betreuungsprozess beteiligten Fachpersonen dar. Bei vulnerablen Angehörigen braucht es eine spezifische Expertise während und nach dem ICU-Aufenthalt. Diese Personengruppen haben ein besonders hohes Risiko, Symptome einer posttraumatischen Belastungsstörung zu erleben. Hier bedarf es gezielter Maßnahmen, um eine personenzentrierte und vulnerabilitätsmindernde Begleitung zu ermöglichen.

## Personenzentrierte Angehörigenbegleitung

Die Bedeutung einer personenzentrierten Angehörigenbegleitung ist in den letzten Jahren stark in den klinischen und wissenschaftlichen Fokus gerückt [1, 2], zum einen wegen der großen Bedeutung für die kritisch kranken Patient*innen und zum anderen wegen der Belastung der Angehörigen in der schwierigen Situation. Die Belastung von besonders vulnerablen Angehörigen wie Kindern und Jugendlichen, hochaltrigen Menschen, sozioökonomisch benachteiligten Personen, ethnischen Minderheiten sowie Menschen mit chronischen oder besonderen medizinischen Bedürfnissen wurden bisher kaum untersucht, obwohl sie zum Familiensystem kritisch kranker Patient*innen gehören [3]. Vulnerabilität beinhaltet in diesem Kontext gemäß Baranzke (2020) das Risiko, einen Schaden zu erleiden, die Unfähigkeit, sich selbst davor zu schützen, und das subjektive Erleben dieser Unfähigkeit [4].

Unter einer personenzentrierten Begleitung versteht sich ebenso ein normatives Wertesystem, dass sich Fachpersonen, Teams oder ganze Organisationen subjektiv zu eigen machen können. Dies basierend auf Werten wie Verständnis, Respekt und Recht auf Selbstbestimmung [5]. Auf der ICU werden kritisch kranke Patient*innen betreut; dabei definiert sich nicht nur deren Persönlichkeit durch die Krankheit/den schweren Unfall, sondern auch ihre Angehörigen sind vom kritischen Ereignis betroffen [6]. Die Angehörigen haben dabei eine wichtige Aufgabe im Genesungsprozess für die kritisch kranken Patient*innen. Sie sind die Stellvertreter*innen, unterstützen bei der Entscheidungsfindung, wirken als Kotherapeut*innen im Empowerment und in der Rehabilitation. Die Angehörigen gelten als wichtiger Teil des Betreuungsteams und sind für das Wohlbefinden und die Nachsorge der kritisch Kranken unabdingbar [7]. Somit gilt bei der personenzentrierten Begleitung: Der Umgang mit den Angehörigen soll vom selben Respekt und der gleichen Wertschätzung geprägt sein, die den kritisch kranken Patient*innen entgegengebracht wird. Dabei gilt es bei vulnerablen Angehörigen, deren Werte und Überzeugungen zu erfahren, mit ihnen zu arbeiten und sie als Persönlichkeit kennenzulernen [6]. Dies bedeutet, gemeinsam mit den Angehörigen herauszufinden, was ihnen in der individuellen Situation wesentlich ist und wie dies erreicht werden kann. Denn Angehörige erleben eine enorme Belastung. Diese Belastung betrifft das persönliche Erleben und hat mitunter psychische, physische, soziale sowie finanzielle Auswirkungen. Diese möglichen Folgen können Symptome eines Post-Intensive-Care-Syndroms - Family (PICS-F) sein [8]. Belastet können Menschen aller Altersgruppen während Tagen, Monaten und Jahren sein [9, 10].

Die Angehörigenbegleitung auf der ICU kann eine große Herausforderung für das Betreuungsteam darstellen. Noch größer kann diese Herausforderung sein, wenn die Begleitung vulnerable Angehörige einschließt. Diese Angehörigen benötigt oft zusätzliche Maßnahmen in einer personenzentrierten und vulnerabilitätsmindernden interprofessionellen Begleitung.

Im folgenden Abschnitt werden die besonderen Herausforderungen der personenzentrierten und vulnerabilitätsmindernden interprofessionellen Begleitung dargestellt. Dabei liegt der Fokus auf den vulnerablen Angehörigen und auf konkreten Unterstützungsmöglichkeiten wie z. B. dem Einsatz digitaler Hilfsmittel.

## Vulnerable Angehörige

Wer gehört dazu? Die Bezeichnung „vulnerable Angehörige“ beschreibt eine Gruppe von Menschen, die anfällig oder verletzlich gegenüber bestimmten Risiken oder Herausforderungen sind.

Besonders herausfordernd ist, wenn vulnerable Gruppen Angehörige von kritisch kranken Patient*innen sind

**Abb. 1:**
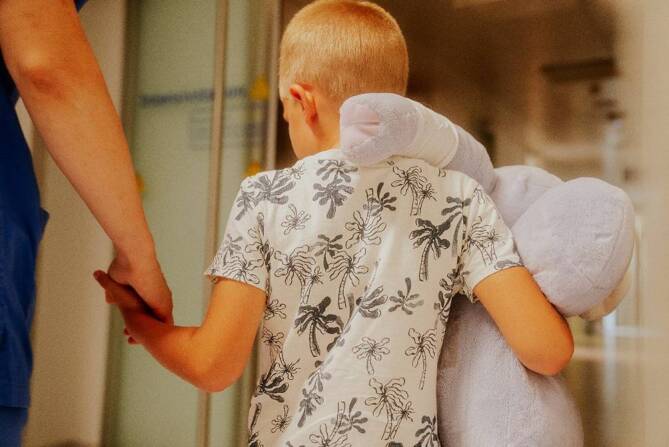
Kinder und Jugendliche als Teil der vulnerablen Angehörigen. (Mit freundl. Genehmigung, © Julia Brunner)

Solche Gruppen können 1. minderjährige Angehörige (Abb. 1), 2. hochaltrige Angehörige, 3. Angehörige mit sozioökonomischer Benachteiligung, 4. Angehörige ethnischer Minderheiten sowie 5. Angehörige mit chronischen Erkrankungen oder mit Behinderungen sein [11]. Diese Angehörige sind von gesundheitlichen, sozialen, wirtschaftlichen oder psychischen Belastungen besonders betroffen und benötigen daher mehr Unterstützung und Schutz. Dies wird umso relevanter, wenn diese vulnerablen Gruppen Angehörige von kritisch kranken Patient*innen sind bzw. werden. Dann braucht es den Bedürfnissen angepasste Unterstützungsangebote.

## Material und Methode

Aufgrund einer Literatursuche und Zusammenfassung von Expert*innenwissen werden im folgenden Abschnitt Herausforderungen und Lösungsansätze für eine personenzentrierte und vulnerabilitätsmindernde Begleitung in der klinischen Praxis beschrieben.

## Ergebnisse

### Minderjährige Angehörige

Minderjährige Angehörige (0–18 Jahre – Kinder und Jugendliche) von kritisch kranken Patient*innen werden oft übersehen und erhalten nicht die notwendige Unterstützung, um mit den emotionalen und psychischen Belastungen umzugehen. Diese Altersgruppe verfügt i. d. R. noch nicht über ausgereifte Coping-Strategien. Manche Minderjährige erleben potenziell traumatische Situationen, wie eine Reanimation oder einen Unfall, hautnah mit. In solchen Fällen kann ein gut begleiteter Besuch auf der ICU eine korrigierende Erfahrung darstellen und dabei helfen, das Erlebte besser zu verarbeiten [12].

Ein entscheidender Faktor für einen erfolgreichen Besuch ist die professionelle Begleitung der Minderjährigen. Häufig bestehen jedoch Vorurteile und Bedenken seitens des Fachpersonals, die zu einer restriktiven Haltung gegenüber Kinderbesuchen auf der ICU führen können. Dazu gehören etwa die Angst vor gegenseitigen Infektionsrisiken oder die Sorge, dass Minderjährige durch den Anblick technischer Geräte, schwerkranker Patient*innen oder der ICU-Umgebung traumatisiert werden könnten. Diese Annahmen tragen dazu bei, dass Minderjährige in vielen Fällen von Besuchen abgehalten werden, obwohl sie durch den Kontakt zu ihren kritisch kranken Angehörigen und durch eine altersgerechte Unterstützung nachweislich profitieren könnten [13].

Die 2022 von der Deutschen Interdisziplinären Vereinigung für Intensiv- und Notfallmedizin (DIVI) veröffentlichten Empfehlungen bieten einen wichtigen Leitfaden für den Einbezug von Minderjährigen auf der ICU. Sie geben dem Fachpersonal praktische Hinweise zur Umsetzung von Kinderbesuchen und helfen, bestehende Barrieren und Vorurteile abzubauen [14, 15].

Ein unterstützendes Angebot für Angehörige stellt die Plattform www.intensivstation.jetzt dar. Sie bietet umfassende, digitale Ressourcen für Minderjährige, deren Angehörige, kritisch kranke Patient*innen sowie Fachpersonen. Altersgerechte Erklärungen, Videos und anschauliche Materialien helfen Minderjährigen, die Umgebung einer ICU besser zu verstehen und ihre Ängste zu reduzieren. Die Plattform stellt zudem praktische Tipps für den ICU-Besuch bereit und gibt Eltern Anregungen, wie sie ihre Kinder in dieser herausfordernden Zeit bestmöglich begleiten können. Durch gezielte Aufklärung, Sensibilisierung des Fachpersonals und altersgerechte Informationsangebote kann die Unterstützung für minderjährige Angehörige deutlich verbessert und deren Rolle im Kontext der ICU gestärkt werden.

### Hochaltrige Angehörige

Der Anteil an hochaltrigen Patient*innen (≥ 80 Jahre) auf der ICU steigt [16, 17]. Dadurch kann auch das Alter der besuchenden Angehörigen steigen. Gerade hochaltrige Menschen sind eine außergewöhnlich heterogene, aber auch vulnerable Zielgruppe, die sich nach finanziellen Möglichkeiten, Bildung, Geschlecht, Migrationsstatus, Zivilstand, sexueller Orientierung und Geschlechtsidentität unterscheidet. Zudem gibt es große Unterschiede in den Bereichen Gesundheit, Lebensstil und Bedürfnisse [18]. Gerade bei einem ICU-Besuch können die Ressourcen dieser Angehörigen genutzt werden. Sie kennen ihr Familienmitglied und liefern wesentliche Informationen. Doch hochaltrige Angehörige leiden auch oft an Einschränkungen wie Seh- und Hörbehinderungen. Teils bedarf es einer gezielten Begleitung, welche insbesondere auf die Sinnesbeeinträchtigung Rücksicht nimmt und darauf abgestimmt ist. So erleichtern Maßnahmen wie eine optimale Beleuchtung, eine lärmreduzierte Umgebung, Privatsphäre, eine vertraute Begleitperson, das Wiederholen von einfachen, verständlichen Informationen und ein Hinweis, welche Fachpersonen zuständig ist, das Zurechtfinden auf der ICU. Es ist wichtig das hochaltrige Angehörige wissen, dass die Fachpersonen nicht nur für die kritisch kranken Patient*innen zuständig sind, sondern auch sie willkommen sind. Im Hinterkopf ist zu halten, dass aus der Geschichte heraus Fachpersonen oft als hohe Autoritätspersonen gesehen wurden und werden. Daher reagieren hochaltrige Menschen mitunter sehr zurückhaltend und wollen sie z. B. nicht mit Fragen belasten.

Des Weiteren ist eine hindernisfreie Zugänglichkeit, eine gute Markierung der räumlichen Gegebenheiten und das Bereitstellen von Sitzgelegenheiten, technischen Hilfsmitteln wie beispielsweise einem Rollator oder Handläufen unabdingbar. Aufgrund der oft nicht einfachen Zugänglichkeit ist es wichtig, dass hochaltrige Angehörige Informationen erhalten, wie sie Auskünfte zu den Patient*innen und zur ICU erhalten können. Viele hochaltrige Angehörige hören schlecht beim Telefonieren und können dadurch Informationen nicht oder missverstehen.

Es ist sinnvoll, den Einsatz digitaler Hilfsmittel im individuellen Kontext zu evaluieren

Auch digitale Informationen werden zunehmend zu einem festen Bestandteil des Alltags von älteren Menschen [19, 20]. Viele nutzen das Internet (74 %) oder besitzen ein Smartphone (64 %) [21]. Jedoch unterscheidet sich die Nutzung markant zwischen Menschen im dritten (65–79 Jahre) und vierten Lebensalter (≥ 80 Jahre). So nutzen beispielsweise 86 % der „jüngeren Älteren“ (65–79 Jahre) das Internet, aber nur 45 % der Menschen ab 80 Jahren [22]. Daher ist es sinnvoll, den Einsatz digitaler Hilfsmittel im individuellen Kontext zu evaluieren [19] sowie niederschwellige Kommunikationsangebote für hochaltrige Angehörige anzubieten.

### Angehörige mit sozioökonomischer Benachteiligung

Sozioökonomisch benachteiligte Menschen stehen ihr gesamtes Leben lang vor Herausforderungen [23], ganz besonders, wenn ihr kritisch kranker Angehöriger auf der ICU hospitalisiert ist. Diese Herausforderungen betreffen sowohl emotionale, kommunikative als auch praktische Aspekte. Eine mögliche Herausforderung ist eine eingeschränkte Gesundheitskompetenz (Health Literacy), was bedeutet, dass diese Angehörigen oft keine angemessenen Entscheidungen für die eigene Gesundheit und jene der Patient*innen treffen können. Es bestehen Schwierigkeiten, medizinische Informationen zu finden, zu verstehen, zu beurteilen und entsprechend zu handeln [24]. Hinzu kommt vielfach eine Unsicherheit im Umgang mit Fachpersonal, was zu Missverständnissen bei Behandlungsprozessen und Prognosen führen kann. Ebenso können finanzielle Belastungen problematisch werden, welche zusätzlich zum bereits engen Budget hinzukommen können, z. B. Reisekosten, Parken oder Einkommensausfälle, wenn die Angehörigen ihre kritisch kranken Patient*innen besuchen. So verhindern restriktive Besuchszeiten, dass Angehörige zu Besuch kommen können, wenn wenig zeitlicher Spielraum etwa durch schwierige Arbeitszeiten oder Betreuungspflichten gegeben ist.

Sozioökonomisch benachteiligte Angehörige haben oft Probleme mit ihrer psychischen und physischen Gesundheit, einen erhöhten Stresslevel, verspüren Müdigkeit, Unsicherheit oder haben Angst vor der ungewohnten ICU-Umgebung. Sie fühlen sich oft nicht verstanden, diskriminiert oder stigmatisiert. Die emotionale Unterstützung von Familie oder Freunden kann eingeschränkt sein, da diese möglicherweise auch in sozial und finanziell schwierigen Verhältnissen leben. Ebenso kann sich der Zugang zu digitalen Hilfsmitteln schwierig gestalten. Als unterstützende Maßnahmen erweisen sich neben einem sensiblen Umgang mit diesen Angehörigen auch das Einbeziehen von Sozialarbeiter*innen. Aufgrund von Schamgefühlen kann es jedoch schwierig sein, Hilfe anzunehmen. Deshalb sollten Informationen zu Unterstützungsmöglichkeiten frühzeitig, z. B. in leicht verständlichen Infomaterialien (Gute Gesundheitsinformationen) vermittelt werden, damit sie verfügbar sind, wenn die Menschen dazu bereit sind. Es braucht eine Bedarfsanalyse zur Förderung der Gesundheitskompetenz bei Angehörigen in schwierigen Lebenslagen durch z. B. sozialberatende Stellen und schnelle (finanzielle) Unterstützungsangebote in Notsituationen, um ICU-Besuche zu ermöglichen.

### Angehörige ethnischer Minderheiten

Verständliche Informationen zu erhalten und in Kontakt zu stehen, ist ein wichtiges Grundbedürfnis von Migrant*innen, deren kritisch kranke Patient*innen auf der ICU betreut werden. Wenn Migrant*innen ihre Angehörigen besuchen, bedarf es eines vertieften Verständnisses der Lebenswelt dieser vulnerablen Angehörigen, damit sie eine gute Begleitung erhalten und nicht resignieren [25]. Migrant*innen sind schon oft vor und während der Migration stark belastenden Lebensumständen ausgesetzt. Die kritische Erkrankung verstärkt diese Umstände weiter. Zudem bestehen oft Sprachprobleme, Integrationsdruck, Arbeitslosigkeit, knappe Wohnmöglichkeiten, größere Familien (viel Besuch) und finanzielle Probleme [26]. Schließlich zeigt sich oft ein unterschiedliches Krankheitsverständnis und eine Unkenntnis über soziokulturelle und religiöse Besonderheiten der vulnerablen Angehörigen [27].

Außerdem bedarf es möglicherweise auch des Einbezugs von Dolmetscher*innen

Eine Unterstützung kann hier eine kultursensible Begleitung bieten, welche die Bedürfnisse erfragt, unterstützt und nach Möglichkeiten umsetzt [28]. Außerdem bedarf es möglicherweise auch des Einbezugs von Dolmetscher*innen, damit sprachliche Barrieren angegangen werden können, um die medizinische Betreuung und die Patient*innensicherheit zu gewähren [26]. Darüber hinaus kann Informationsmaterial (Gute Gesundheitsinformationen) auch in unterschiedlichen Sprachen übersetzt vorgehalten werden.

### Angehörige mit chronischen Erkrankungen oder mit Behinderungen

Angehörige mit chronischen Erkrankungen können zum einen selbst einer schweren Krankheitsbelastung ausgesetzt sein und zum anderen genau dadurch sehr viel Erfahrung haben. Als Angehörige können sie mit verschiedenen Herausforderungen konfrontiert sein. Angehörige mit einem herabgesetzten Immunsystem oder einer infektiösen Krankheit können ggf. überhaupt nicht oder nur unter herausfordernden Bedingungen die ICU aufsuchen. Angehörige mit Bewegungseinschränkungen schaffen es möglicherweise nicht ohne fremde Unterstützung, ihre kritisch kranken Patient*innen zu besuchen. Angehörige mit einer physischen oder psychischen Behinderung benötigen eine Begleitperson und angepasste Kommunikationsstrategien wie z. B. Gebärdensprachdolmetschen. Diese Einschränkungen sind so vielfältig, dass es sich lohnt, in der Anamnese danach zu fragen. Daher sind handlungsrelevante Gesundheitsinformationen vor einem ICU-Besuch für diese Angehörigen sehr relevant, die verständlich gestaltet, evidenzbasiert sind und mögliche Risiken einfach darstellen. Zudem ist es sinnvoll, die Kompetenzen und Ressourcen dieser Angehörigen zu nutzen, indem ein individuelles und niederschwelliges Vorgehen erarbeitet wird, wie sie den Genesungsprozess der kritisch kranken Patient*innen unterstützen können (z. B. Videoaufnahmen usw.) [29, 30].

### Maßnahmen zur Unterstützung

Im Folgenden sind zusammenfassend Maßnahmen angeführt, die es ermöglichen, vulnerable Angehörige zu identifizieren und diesen einen ICU-Besuch unter den bestmöglichen Bedingungen ermöglichen.

#### Haltung, Kultur und Leadership

Die Haltung des ICU-Managements ermöglicht es, dass sich eine offene Kultur und eine Sensibilität gegenüber vulnerablen Angehörigen entwickeln kann. Das bedeutet nicht, dass es allein die Aufgabe des Managements ist, sondern vielmehr, dass dieses offen ist, sich selbst als ein Vorbild wahrnimmt und die Initiativen der Mitarbeiter*innen zu einer offenen, lernenden und wertschätzenden Kultur unterstützt. So ist es sinnvoll, bereits initial in der Angehörigenanamnese zu erfassen, welche Menschen zur Patientin bzw. zum Patienten gehören, in welcher Beziehung diese zueinanderstehen und ob es ein besonderes Unterstützungsangebot braucht.

#### Unterstützungsangebote in der Praxis

Es gibt eine Vielzahl an Angeboten, die für vulnerablere Angehörige eine große Unterstützung sein können. Dazu zählen Schulungen, Microlearning-Angebote und Standard Operation Procedures (SOP), wo Maßnahmen für alle am Behandlungsprozess beteiligten Personen nachvollziehbar beschrieben sind, Besuchszeiten, die sich an den Bedürfnissen der vulnerablen Angehörigen orientieren, oder definierte und geschulte Teammitglieder, die sich mit der Angehörigenbegleitung befassen, schriftliche Informationsangebote in unterschiedlichen Sprachen, u. a. in lesefreundlichem Format (einfache oder leichte Sprache), strukturelle Angebote (z. B. Verpflegung) sowie ein gut ausgestatteter Wartebereich.

Zudem bedarf es einer Förderung der multidisziplinären Zusammenarbeit und digitaler Angebote. Die Nutzung von digitalen Unterstützungsangeboten kann für vulnerable Angehörige einen Vorteil darstellen, jedoch nicht für alle in gleichem Maß. Angebote wie Webseiten mit leicht verständlichem Inhalt in unterschiedlichen Sprachen, Videotelefonie, Videodolmetschen, digitale Intensivtagebücher und ggf. Online-Selbsthilfegruppen oder Follow-up-Angebote können hier beispielhaft genannt werden. Bei allen Bemühungen um eine vermehrte Digitalisierung bei verschiedenen vulnerablen Angehörigen sollte nicht außer Acht gelassen werden, dass einzelne Angehörige (z. B. Menschen mit geistiger Behinderung, starker kognitiver Einschränkung) wenig online sein werden oder andere sich bewusst gegen digitale Angebote entscheiden. Weiter gilt es zu bedenken, dass bei der Entwicklung dieser Unterstützungsangebote vulnerable Angehörige miteinbezogen werden sollten.

Es ist nicht nötig, alles für alle anzubieten, es ist jedoch möglich, eine offene Haltung einzunehmen

Es ist nicht nötig, alles für alle anzubieten, es ist jedoch möglich, eine Haltung einzunehmen, die es zulässt, dass Bedürfnisse offen kommuniziert werden und dass vulnerable Angehörige so die bestmöglich verfügbare Unterstützung erhalten.

Eine Zusammenfassung der Gruppen von vulnerablen Angehörigen sowie von deren Herausforderungen und Unterstützungsmöglichkeiten ist in Tab. 1 ersichtlich.

**Tab. 1:** Vulnerable Populationen und deren Herausforderungen inkl. konkrete Maßnahmen für die Praxis

Vulnerable Population	Herausforderung im Kontext Intensivstation	Unterstützung
Minderjährige Angehörige (Kinder, Jugendliche)	Sorge um Traumatisierung, Sorge um wechselseitige Infektionsgefahr, fehlendes Wissen um kognitive Entwicklung bei Kindern und Jugendlichen, fehlende Erfahrungen	Empfehlung der DIVI zum Einbezug von Minderjährigen in der Intensiv- und Notfallmedizin, Gute Gesundheitsinformationen, www.intensivstation.jetzt/kinder, Intensivtagebuch mit Fotos, (Online‑)Intensivtagebücher, entsprechend ausgestatteter Wartebereich, adäquate Verpflegungsmöglichkeiten, Videotelefonie, Einbezug von Psycholog*innen
Hochaltrige Menschen	Sorge, eine Last darzustellen, Einschränkungen Seh- und Hörvermögen, Einschränkungen in Kognition und Mobilität, eingeschränkte Zugänglichkeit, Architektur	Gute Gesundheitsinformationen, Begleitdienste/vertraute Begleitperson, optimale Beleuchtung, eine lärmreduzierte Umgebung, Privatsphäre, Wiederholen von einfachen, verständlichen Informationen, Digitalisierung mit der individuellen Person klären, (Online‑)Intensivtagebücher, ausreichend Sitzmöglichkeiten, adäquate Verpflegungsmöglichkeiten, Videotelefonie
Menschen mit sozioökonomisch Benachteiligung	Scham, mangelnde finanzielle und zeitliche Ressourcen, geringe Gesundheitskompetenz	Gute Gesundheitsinformationen, flexible Besuchszeiten, finanzielle Unterstützung für Notsituationen, um Besuche zu ermöglichen, adäquate Verpflegungsmöglichkeiten, (Online‑)Intensivtagebücher, Beratung durch Sozialarbeiter*innen
Menschen ethnischer Minderheiten	Kulturelle Unterschiede, Sprachbarriere	Gute Gesundheitsinformationen, Übersetzungs- und Dolmetscherdienste, Videodolmetscher*innen, https://www.gesundheit.gv.at/gesundheitsleistungen/gesundheitsfoerderung/migration-gesundheitsfoerderung.html
Menschen mit chronischen Erkrankungen oder mit Behinderungen	Sorge um wechselseitige Infektionsgefahr, Information- und Kommunikationsmängel, risikobehaftetes Verhalten	Gute Gesundheitsinformationen, Videotelefonie, Beratung durch Sozialarbeiter*innen oder Patient*innenlotsen, flexible Besuchszeiten oder finanzielle Hilfen für Reisekosten, Schulung des Personals zu Diversität und Sensibilität gegenüber sozioökonomischen Bedürfnissen, (Online‑)Intensivtagebücher, sonstige Begleitdienste

### Limitationen

Dieser Artikel kann nur exemplarisch die Herausforderungen der vulnerablen Angehörigen aufzeigen. Er soll zur adäquaten Begleitung vulnerabler Angehöriger einen Denkanstoß geben. Bisher gibt es wenig Untersuchungen dazu, künftig bedarf es weiterer Forschung und Erfahrungen im Bereich dieser wichtigen Angehörigenpopulation.

## Ausblick

Angehörige von kritisch kranken Patient*innen erleben oft eine Lebenskrise während oder nach dem ICU-Aufenthalt. Besonders trifft dies auf vulnerable Angehörige zu, wie Kinder und Jugendliche, hochaltrige Angehörige, sozioökonomisch benachteiligte Angehörige, ethnische Minderheiten sowie Angehörige mit chronischen oder besonderen medizinischen Bedürfnissen. Eine Sensibilisierung der Fachpersonen in Bezug auf diese besondere Thematik kann zu einer verbesserten Begleitung führen. Konkrete Unterstützungsangebote reichen von institutionell-strukturellen Angeboten wie flexiblen Besuchszeiten, optimierten Wartebereichen inklusive einer adäquaten Verpflegung oder finanzieller Unterstützung bis hin zu Vermittlung von Informationen (z. B. Unterstützung durch Sozialdienste oder bestehende Plattformen wie unsere Website www.intensivstation.jetzt). Auch Videotelefonie oder auf künstlicher Intelligenz (KI) basierende digitale Weiterentwicklungen bieten eine große Chance. Gerade bei sprachlichen Barrieren oder Hör‑/Seheinschränkungen. Mit Empathie und unterstützenden Angeboten kann die Vulnerabilität gemindert werden und die Resilienz der Betroffenen verbessert werden.

## Fazit für die Praxis

- Angehörige sind zumeist eine wichtige Ressource für die Betroffenen.
- Angehörige haben generell ein sehr großes Risiko, selbst Symptome einer posttraumatischen Belastungsstörung zu erleben.
- Angehörige können zu der Gruppe vulnerabler Menschen gehören, wenn sie z. B. sehr jung oder sehr alt sind, unter chronischen Erkrankungen oder Behinderungen leiden, ethnischen Minderheiten angehören oder sozioökonomisch benachteiligte Personen sind.
- Vulnerable Angehörige haben ein erhöhtes Risiko, belastende Langzeitfolgen zu erleiden.
- Die Grundhaltung der Fachpersonen auf der Intensivstation (ICU) soll darin bestehen, diese Angehörigen zu identifizieren und entsprechende Angebote bereitzuhalten, um diese Menschen zu unterstützen und eine individuell angepasste Partizipation zu ermöglichen.

## Abkürzungen

ICU: Intensive Care Unit
ÖGIAIN: Österreichische Gesellschaft für Internistische und Allgemeine Intensivmedizin und Notfallmedizin
PICS‑F: Post-Intensive-Care-Syndrom - Family
SOP: Standard Operating Procedure
